# Bis(9-amino­acridinium) bis­(pyridine-2,6-dicarboxyl­ato)zincate(II) trihydrate

**DOI:** 10.1107/S1600536812005764

**Published:** 2012-03-03

**Authors:** M. Mirzaei, H. Eshtiagh-Hosseini, E. Eydizadeh, Z. Yousefi, K. Molčanov

**Affiliations:** a Department of Chemistry, Ferdowsi University of Mashhad, 917791436 Mashhad, Iran; b Laboratory of Chemical Crystallography and Biocrystallography, Department of Physical Chemistry, Rudjer Bošković Institute, Bijenička 54, HR-10000, Zagreb, Croatia

## Abstract

In the title compound, (C_13_H_11_N_2_)_2_[Zn(C_7_H_3_NO_4_)_2_]·3H_2_O, the Zn^II^ ion is six-coordinated with the N_4_O_2_ donor set being a distorted octa­hedron through two almost perpendicular (r.m.s. deviation of ligand atoms from the mean plane is 0.057 Å) tridentate pyridine-2,6-dicarboxyl­ate ligands [dihedral angle between the ligands = 86.06 (4)°]. The charge is compensated by two 9-amino­acridinium cations protonated on the ring N atom. A variety of inter­molecular contacts, such as ion–ion, N—H⋯O and O—H⋯O hydrogen bonds, and π–π stacking [centroid–centroid distances = 3.4907 (9)–4.1128 (8) Å], between cations and between anions, play important roles in the formation of the three-dimensional network.

## Related literature

For the behaviour of 9-amino­acridine in coordination compounds see: Derikvand *et al.* (2010 ▶); Eshtiagh-Hosseini, Mirzaei, Eydizadeh *et al.* (2011 ▶). For a brief review of the pyridine­dicarboxyl­ate family of ligands, see: Mirzaei *et al.* (2011 ▶). For related structures, see: Aghabozorg *et al.* (2008 ▶); Derikvand *et al.* (2010 ▶); Eshtiagh-Hosseini, Yousefi, Mirzaei *et al.* (2010 ▶); Eshtiagh-Hosseini, Mirzaei, Eydizadeh *et al.* (2011 ▶); Eshtiagh-Hosseini, Mirzaei, Yousefi *et al.* (2011 ▶); Eshtiagh-Hosseini, Yousefi, Shafiee *et al.* (2010 ▶); Harrison *et al.* (2006 ▶); MacDonald *et al.* (2000 ▶); Park *et al.* (2007 ▶); Tabatabaee *et al.* (2009 ▶).
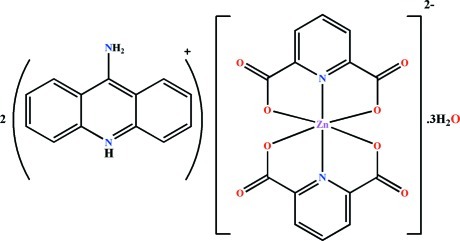


## Experimental

### 

#### Crystal data

(C_13_H_11_N_2_)_2_[Zn(C_7_H_3_NO_4_)_2_]·3H_2_O
*M* *_r_* = 840.1
Triclinic, 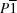

*a* = 10.8763 (3) Å
*b* = 13.3802 (3) Å
*c* = 13.9920 (4) Å
α = 102.359 (2)°
β = 103.585 (2)°
γ = 105.137 (2)°
*V* = 1826.44 (8) Å^3^
*Z* = 2
Cu *K*α radiation
μ = 1.57 mm^−1^
*T* = 293 K
0.1 × 0.1 × 0.1 mm

#### Data collection

Xcalibur Nova R CCD diffractometer
Absorption correction: multi-scan (*CrysAlis PRO*; Agilent, 2011 ▶) *T*_min_ = 0.786, *T*_max_ = 1
18061 measured reflections
7540 independent reflections
6901 reflections with *I* > 2σ(*I*)
*R*_int_ = 0.023

#### Refinement

*R*[*F* ^2^ > 2σ(*F* ^2^)] = 0.032
*wR*(*F* ^2^) = 0.091
*S* = 1.03
7540 reflections
547 parameters
9 restraints
H atoms treated by a mixture of independent and constrained refinement
Δρ_max_ = 0.23 e Å^−3^
Δρ_min_ = −0.33 e Å^−3^

### 

Data collection: *CrysAlis PRO* (Agilent, 2011 ▶); cell refinement: *CrysAlis PRO*; data reduction: *CrysAlis PRO*; program(s) used to solve structure: *SHELXS97* (Sheldrick, 2008 ▶); program(s) used to refine structure: *SHELXL97* (Sheldrick, 2008 ▶); molecular graphics: *ORTEP-3 for Windows* (Farrugia, 1997 ▶); software used to prepare material for publication: *WinGX* (Farrugia, 1999 ▶).

## Supplementary Material

Crystal structure: contains datablock(s) global, I. DOI: 10.1107/S1600536812005764/mw2050sup1.cif

Structure factors: contains datablock(s) I. DOI: 10.1107/S1600536812005764/mw2050Isup2.hkl

Additional supplementary materials:  crystallographic information; 3D view; checkCIF report

## Figures and Tables

**Table 1:** Hydrogen-bond geometry (Å, °)

*D*—H⋯*A*	*D*—H	H⋯*A*	*D*⋯*A*	*D*—H⋯*A*
N3—H3*A*⋯O9	0.86	1.89	2.7013 (18)	157
N4—H4*A*⋯O8^i^	0.86	1.98	2.8005 (18)	160
N4—H4*B*⋯O3^ii^	0.86	2.21	2.9589 (19)	145
N5—H5*A*⋯O4	0.86	1.88	2.7351 (19)	174
N6—H6*A*⋯O2^iii^	0.86	2.21	2.9763 (18)	148
N6—H6*B*⋯O11	0.86	2.10	2.899 (2)	154
O9—H9*A*⋯O8^iv^	0.93 (3)	1.85 (3)	2.768 (2)	170 (2)
O9—H9*B*⋯O10	0.89 (2)	1.86 (2)	2.745 (2)	173 (2)
O10—H10*A*⋯O2^v^	0.94 (2)	1.91 (2)	2.838 (2)	175 (2)
O10—H10*B*⋯O2	0.95 (3)	1.91 (3)	2.830 (2)	161 (2)
O11—H11*A*⋯O6^vi^	0.90 (3)	1.93 (3)	2.825 (2)	176 (3)
O11—H11*B*⋯O1^iii^	0.90 (2)	1.99 (2)	2.8869 (19)	174 (2)

Symmetry codes: (i) 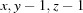; (ii) 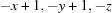; (iii) 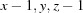; (iv) 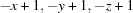; (v) 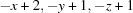; (vi) 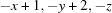.

**Fig. 1.**
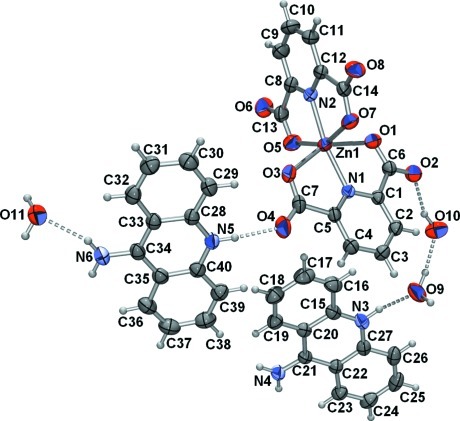
An ORTEP view of the asymmetric unit of the title compound with numbering of the non-hydrogen atoms (probability 50%)

**Fig. 2.**
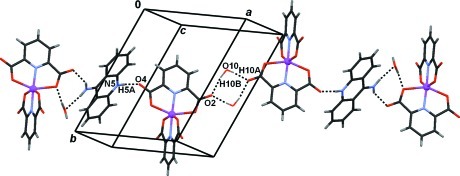
The chain formed by the anionic complex and the water molecules. Zinc ions are depicted as spheres of arbitrary radii.

**Fig. 3.**
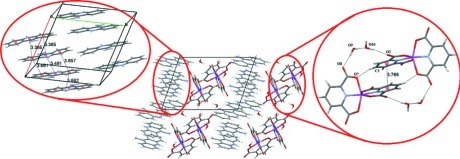
The π–π stacking interactions between the cations and between the anions. (Cg1 and Cg2: N1, C1, C2, C3, C4 and C5; Cg8: C15, C16, C17, C18, C19 and C20; Cg3 and Cg6: N3, C15, C20, C21, C22 and C27; Cg4 and Cg5: C22, C23, C24, C25, C26 and C27; Cg8: C35, C36, C37, C38, C39 and C40;Cg9: N5, C28, C33, C34, C35 and C40; Cg10: C28, C29, C30, C31, C32 and C33)

## supplementary crystallographic information

### Comment

The pyridinedicarboxylate family of ligands has attracted much attention in coordination and supramolecular chemistry because of the versatile coordination modes and variety of inter- and intramolecular interactions (Mirzaei *et al.*, 2011). Among different derivatives of pyridinedicarboxylate, pyridine-2,6-dicarboxylic acid (pydcH_2_), also called dipicolinic acid (H_2_pdic), has been widely considered because of its high symmetry and bioactive properties. The most common coordination mode of (pydc)^2-^ is as a tridentate ligand *via* N and two carboxylate groups that can be coordinated to a metal in a *meridional* fashion (Eshtiagh-Hosseini, Mirzaei, Yousefi *et al.*, 2011; Park *et al.*, 2007).

So far, our group has reported several coordination compounds bearing the (pydc)^2-^ ligand with different heterocyclic cations prepared by proton transfer methodology (Eshtiagh-Hosseini, Yousefi, Mirzaei *et al.*, 2010; Eshtiagh-Hosseini, Yousefi, Shafiee *et al.*, 2010).

In this contribution, we have synthesized and characterized a new coordination compound with (pydc)^2-^ coordinated to Zn^II^ and protonated 9-aminoacridine as the cation which is formulated as (9aaH)_2_[Zn(pydc)_2_].3H_2_O.

The asymmetric unit of the title compound comprises a dianionic complex, [Zn(pydc)_2_]^2-^, two 9aaH^+^ cations and three water molecules (Fig. 1). In the anionic complex, Zn^II^ is six-coordinated *via* two (pydc)^2-^ ions with the ZnN_2_O_4_ donor set in a distorted octahedral geometry. The two (pydc)^2-^ moieties are almost perpendicular to each other (the angle between the mean ligand planes (rms deviation of ligand atoms from the mean plane is 0.057 Å) intersecting at Zn1 is 86.62 (2)°). Bond lengths and angles are comparable with those in similar structures (Tabatabaee *et al.*, 2009; MacDonald *et al.*, 2000; Aghabozorg *et al.*, 2008; Harrison *et al.*, 2006). Recently, our group reported a similar compound with Mn(II) as a metal center which has the same stochiometery as the title compound (Eshtiagh-Hosseini, Mirzaei, Eydizadeh *et al.*, 2011). Binding of the H_2_O molecules to the anionic complex and the 9aaH^+^ cations occur *via* N—H···O and O—H···O hydrogen bonds creating two different motifs with graph sets *R*^4^_2_(8) and *R*_3_^3^(9) (Fig. 2). In Fig. 3, a packing diagram of the title compound viewed down the *b* axis is shown in which a variety of intermolecular contacts can be observed. The most significant additional interactions are π-π stacking between (pydc)^2-^ ligands in adjacent anions and between sets of 9aaH^+^ cations (Fig. 3).

### Experimental

To 5 mL of an aqeous solution of pydcH_2_ (0.026 g, 0.15 mmol), 5 mL of a methanolic solution of 9aa (0.030 g,0.15 mmol) was added dropwise. Then, powdered ZnCl_2_.2H_2_O (0.011 g, 0.075 mmol) was added and the resulting solution was heated and stirred for 3 hrs at 60°C. Yellow crystals were obtained by slow evaporation of the solvent at room temperature after 3 days.

### Refinement

A full-matrix least-squares refinement implemented in the *SHELXL97* (Sheldrick, 2008) was used. All non-H atoms were refined anisotropically. The H atoms were positioned geometrically and allowed to ride on their parent atoms, with C—H = 0.93 Å and 0.97 Å for C and 0.86 Å for N atom and *U_iso_*(H) = 1.2 *U_eq_*(C,*N*). The H atoms of water were located in difference map and refined with the following restraints: O—H = 0.95 (2) Å and H···H = 1.50 (4) Å (total of 9 restraints were used).

### Crystal data

**Table:**

(C_13_H_11_N_2_)_2_[Zn(C_7_H_3_NO_4_)_2_]·3H_2_O	*Z* = 2
*M**_r_* = 840.1	*F*(000) = 868
Triclinic, *P*1	*D*_x_ = 1.528 Mg m^−^^3^
Hall symbol: -P 1	Cu *K*α radiation, λ = 1.54184 Å
*a* = 10.8763 (3) Å	Cell parameters from 12486 reflections
*b* = 13.3802 (3) Å	θ = 3.4–75.7°
*c* = 13.9920 (4) Å	µ = 1.57 mm^−^^1^
α = 102.359 (2)°	*T* = 293 K
β = 103.585 (2)°	Prism, yellow
γ = 105.137 (2)°	0.1 × 0.1 × 0.1 mm
*V* = 1826.44 (8) Å^3^	

### Data collection

**Table:**

Xcalibur Nova R CCD diffractometer	6901 reflections with *I* > 2σ(*I*)
ω scans	*R*_int_ = 0.023
Absorption correction: multi-scan (*CrysAlis PRO*; Agilent, 2011)	θ_max_ = 75.9°, θ_min_ = 3.4°
*T*_min_ = 0.786, *T*_max_ = 1	*h* = −13→13
18061 measured reflections	*k* = −15→16
7540 independent reflections	*l* = −17→14

### Refinement

**Table:**

Refinement on *F*^2^	9 restraints
Least-squares matrix: full	H atoms treated by a mixture of independent and constrained refinement
*R*[*F*^2^ > 2σ(*F*^2^)] = 0.032	*w* = 1/[σ^2^(*F*_o_^2^) + (0.0487*P*)^2^ + 0.2947*P*] where *P* = (*F*_o_^2^ + 2*F*_c_^2^)/3
*wR*(*F*^2^) = 0.091	(Δ/σ)_max_ = 0.001
*S* = 1.03	Δρ_max_ = 0.23 e Å^−^^3^
7540 reflections	Δρ_min_ = −0.33 e Å^−^^3^
547 parameters	

### Special details

**Table:**

Geometry. All e.s.d.'s (except the e.s.d. in the dihedral angle between two l.s. planes) are estimated using the full covariance matrix. The cell e.s.d.'s are taken into account individually in the estimation of e.s.d.'s in distances, angles and torsion angles; correlations between e.s.d.'s in cell parameters are only used when they are defined by crystal symmetry. An approximate (isotropic) treatment of cell e.s.d.'s is used for estimating e.s.d.'s involving l.s. planes.

### Fractional atomic coordinates and isotropic or equivalent isotropic displacement parameters (Å^2^)

**Table:**

	*x*	*y*	*z*	*U*_iso_*/*U*_eq_	
Zn1	0.63843 (2)	0.757062 (15)	0.410290 (14)	0.04351 (8)	
N1	0.56573 (12)	0.59395 (9)	0.37952 (8)	0.0339 (2)	
N2	0.71182 (13)	0.91958 (9)	0.46845 (9)	0.0389 (2)	
O1	0.79818 (13)	0.71221 (9)	0.51306 (9)	0.0519 (3)	
O2	0.85048 (12)	0.56753 (10)	0.53916 (9)	0.0507 (3)	
O7	0.53943 (14)	0.79380 (9)	0.52860 (9)	0.0514 (3)	
O3	0.44757 (13)	0.71287 (9)	0.29115 (8)	0.0494 (3)	
O5	0.75856 (15)	0.80175 (10)	0.31737 (9)	0.0560 (3)	
O8	0.52661 (14)	0.93159 (10)	0.64241 (9)	0.0555 (3)	
O6	0.90581 (15)	0.94355 (12)	0.30107 (11)	0.0626 (3)	
C6	0.77303 (15)	0.61256 (12)	0.49985 (10)	0.0394 (3)	
O4	0.26883 (14)	0.56750 (12)	0.19614 (11)	0.0649 (4)	
C5	0.44187 (14)	0.54306 (11)	0.31581 (10)	0.0361 (3)	
C14	0.57104 (17)	0.89226 (12)	0.57460 (10)	0.0414 (3)	
C1	0.63454 (14)	0.54097 (11)	0.42913 (9)	0.0339 (3)	
C2	0.57841 (16)	0.43143 (12)	0.41608 (11)	0.0397 (3)	
H2	0.6266	0.3947	0.4504	0.048*	
C12	0.67212 (16)	0.96945 (11)	0.54266 (10)	0.0397 (3)	
C8	0.80023 (16)	0.97360 (12)	0.43042 (11)	0.0424 (3)	
C7	0.37918 (16)	0.61393 (13)	0.26292 (11)	0.0436 (3)	
C13	0.82615 (18)	0.90143 (13)	0.34225 (12)	0.0464 (3)	
C11	0.7210 (2)	1.08125 (13)	0.58237 (12)	0.0503 (4)	
H11	0.6917	1.1166	0.6328	0.06*	
C3	0.44891 (17)	0.37785 (12)	0.35081 (12)	0.0441 (3)	
H3	0.4088	0.3045	0.3415	0.053*	
C4	0.37925 (16)	0.43381 (13)	0.29935 (11)	0.0432 (3)	
H4	0.2925	0.3988	0.2549	0.052*	
C9	0.8561 (2)	1.08539 (14)	0.46910 (14)	0.0544 (4)	
H9	0.92	1.1236	0.4445	0.065*	
C10	0.8145 (2)	1.13889 (14)	0.54521 (14)	0.0590 (4)	
H10	0.8496	1.2139	0.5714	0.071*	
H11A	−0.024 (3)	0.9280 (18)	−0.289 (2)	0.097 (10)*	
H11B	−0.120 (2)	0.8243 (18)	−0.3509 (14)	0.076 (7)*	
H9A	0.620 (2)	0.160 (2)	0.339 (2)	0.098 (9)*	
H9B	0.749 (2)	0.2507 (17)	0.3719 (15)	0.073 (7)*	
H10B	0.866 (3)	0.4303 (18)	0.480 (2)	0.101 (10)*	
H10A	0.971 (2)	0.383 (2)	0.466 (2)	0.100 (10)*	
N5	0.14315 (12)	0.60732 (10)	0.02168 (9)	0.0392 (2)	
H5A	0.1775	0.5916	0.0761	0.047*	
C28	0.10576 (13)	0.69735 (12)	0.03227 (11)	0.0372 (3)	
C35	0.07187 (15)	0.56409 (13)	−0.16288 (11)	0.0420 (3)	
N6	−0.01511 (16)	0.68630 (13)	−0.23874 (10)	0.0527 (3)	
H6A	−0.0239	0.6464	−0.2986	0.063*	
H6B	−0.0378	0.7437	−0.2329	0.063*	
O11	−0.08439 (18)	0.86907 (13)	−0.28590 (11)	0.0707 (4)	
C33	0.04982 (13)	0.72633 (12)	−0.05563 (11)	0.0381 (3)	
C29	0.12309 (16)	0.76160 (14)	0.13159 (11)	0.0447 (3)	
H29	0.1597	0.7421	0.189	0.054*	
C32	0.01335 (15)	0.82143 (13)	−0.03963 (13)	0.0453 (3)	
H32	−0.024	0.8421	−0.096	0.054*	
C40	0.12830 (14)	0.54121 (12)	−0.07164 (11)	0.0396 (3)	
C39	0.17044 (17)	0.44950 (13)	−0.07668 (13)	0.0484 (3)	
H39	0.2069	0.4342	−0.0166	0.058*	
C31	0.03218 (17)	0.88292 (14)	0.05684 (14)	0.0501 (4)	
H31	0.0092	0.9458	0.0658	0.06*	
C30	0.08634 (18)	0.85207 (15)	0.14347 (13)	0.0506 (4)	
H30	0.097	0.8938	0.2091	0.061*	
C34	0.03351 (14)	0.65921 (13)	−0.15526 (11)	0.0411 (3)	
C37	0.1025 (2)	0.40490 (16)	−0.26117 (14)	0.0606 (4)	
H37	0.0947	0.3594	−0.3242	0.073*	
C36	0.0601 (2)	0.49258 (15)	−0.25790 (13)	0.0556 (4)	
H36	0.0229	0.5057	−0.319	0.067*	
C38	0.15744 (19)	0.38316 (15)	−0.17021 (16)	0.0573 (4)	
H38	0.1855	0.3229	−0.1733	0.069*	
C27	0.67785 (14)	0.08240 (13)	0.05915 (11)	0.0407 (3)	
N3	0.66525 (13)	0.17238 (11)	0.11676 (9)	0.0439 (3)	
H3A	0.6948	0.1886	0.1827	0.053*	
N4	0.54649 (14)	0.10780 (11)	−0.19805 (9)	0.0443 (3)	
H4B	0.5127	0.1504	−0.225	0.053*	
H4A	0.5574	0.0536	−0.2366	0.053*	
C23	0.65311 (16)	−0.03901 (13)	−0.10529 (13)	0.0453 (3)	
H23	0.6285	−0.0575	−0.1767	0.054*	
C21	0.58166 (13)	0.12563 (11)	−0.09735 (10)	0.0359 (3)	
C20	0.56319 (14)	0.21631 (12)	−0.03328 (10)	0.0372 (3)	
C15	0.60783 (15)	0.23768 (12)	0.07443 (11)	0.0408 (3)	
C22	0.63677 (14)	0.05541 (12)	−0.04935 (11)	0.0375 (3)	
C19	0.49415 (16)	0.28113 (13)	−0.07401 (12)	0.0446 (3)	
H19	0.4608	0.2665	−0.1449	0.054*	
C24	0.70489 (18)	−0.10350 (14)	−0.05529 (16)	0.0542 (4)	
H24	0.714	−0.1659	−0.0929	0.065*	
C25	0.74415 (17)	−0.07573 (16)	0.05241 (16)	0.0560 (4)	
H25	0.7788	−0.1203	0.0855	0.067*	
C26	0.73230 (16)	0.01550 (15)	0.10917 (14)	0.0512 (4)	
H26	0.76	0.0337	0.1806	0.061*	
C18	0.4757 (2)	0.36484 (15)	−0.01080 (15)	0.0546 (4)	
H18	0.4294	0.4065	−0.0387	0.066*	
C17	0.5266 (2)	0.38842 (15)	0.09648 (15)	0.0593 (4)	
H17	0.5163	0.4473	0.1391	0.071*	
C16	0.59065 (19)	0.32654 (15)	0.13869 (13)	0.0530 (4)	
H16	0.6232	0.3426	0.2098	0.064*	
O9	0.68488 (17)	0.20134 (14)	0.31754 (10)	0.0723 (4)	
O10	0.88557 (15)	0.36441 (12)	0.47389 (13)	0.0688 (4)	

### Atomic displacement parameters (Å^2^)

**Table:**

	*U*^11^	*U*^22^	*U*^33^	*U*^12^	*U*^13^	*U*^23^
Zn1	0.06383 (14)	0.02983 (11)	0.03874 (11)	0.01721 (9)	0.01691 (9)	0.01034 (8)
N1	0.0428 (6)	0.0324 (5)	0.0296 (5)	0.0167 (5)	0.0112 (4)	0.0100 (4)
N2	0.0525 (7)	0.0318 (5)	0.0337 (5)	0.0155 (5)	0.0126 (5)	0.0110 (4)
O1	0.0553 (7)	0.0368 (5)	0.0517 (6)	0.0123 (5)	0.0013 (5)	0.0098 (5)
O2	0.0455 (6)	0.0502 (6)	0.0523 (6)	0.0214 (5)	0.0025 (5)	0.0135 (5)
O7	0.0785 (8)	0.0352 (5)	0.0488 (6)	0.0195 (5)	0.0323 (6)	0.0138 (4)
O3	0.0652 (7)	0.0457 (6)	0.0444 (5)	0.0271 (5)	0.0125 (5)	0.0210 (5)
O5	0.0796 (9)	0.0433 (6)	0.0523 (6)	0.0203 (6)	0.0349 (6)	0.0126 (5)
O8	0.0712 (8)	0.0487 (6)	0.0445 (6)	0.0158 (6)	0.0273 (6)	0.0037 (5)
O6	0.0747 (9)	0.0645 (8)	0.0610 (7)	0.0219 (7)	0.0370 (7)	0.0262 (6)
C6	0.0440 (7)	0.0399 (7)	0.0354 (6)	0.0174 (6)	0.0094 (5)	0.0117 (5)
O4	0.0563 (7)	0.0733 (9)	0.0597 (7)	0.0205 (6)	−0.0035 (6)	0.0330 (7)
C5	0.0422 (7)	0.0390 (7)	0.0300 (6)	0.0169 (6)	0.0106 (5)	0.0125 (5)
C14	0.0568 (8)	0.0387 (7)	0.0320 (6)	0.0197 (6)	0.0146 (6)	0.0110 (5)
C1	0.0417 (7)	0.0347 (6)	0.0299 (5)	0.0183 (5)	0.0118 (5)	0.0107 (5)
C2	0.0498 (8)	0.0378 (7)	0.0393 (6)	0.0219 (6)	0.0151 (6)	0.0157 (5)
C12	0.0522 (8)	0.0340 (7)	0.0318 (6)	0.0166 (6)	0.0094 (6)	0.0085 (5)
C8	0.0520 (8)	0.0388 (7)	0.0390 (7)	0.0159 (6)	0.0133 (6)	0.0160 (6)
C7	0.0498 (8)	0.0520 (9)	0.0372 (7)	0.0243 (7)	0.0126 (6)	0.0212 (6)
C13	0.0576 (9)	0.0475 (8)	0.0408 (7)	0.0204 (7)	0.0188 (7)	0.0186 (6)
C11	0.0702 (11)	0.0367 (7)	0.0395 (7)	0.0173 (7)	0.0145 (7)	0.0055 (6)
C3	0.0528 (8)	0.0340 (7)	0.0462 (7)	0.0125 (6)	0.0153 (6)	0.0150 (6)
C4	0.0427 (7)	0.0432 (8)	0.0395 (7)	0.0104 (6)	0.0085 (6)	0.0128 (6)
C9	0.0640 (10)	0.0408 (8)	0.0532 (9)	0.0069 (7)	0.0187 (8)	0.0153 (7)
C10	0.0770 (12)	0.0323 (7)	0.0558 (9)	0.0079 (8)	0.0163 (9)	0.0064 (7)
N5	0.0397 (6)	0.0423 (6)	0.0369 (6)	0.0156 (5)	0.0086 (5)	0.0153 (5)
C28	0.0324 (6)	0.0401 (7)	0.0391 (7)	0.0114 (5)	0.0099 (5)	0.0134 (5)
C35	0.0380 (7)	0.0430 (7)	0.0393 (7)	0.0092 (6)	0.0079 (5)	0.0101 (6)
N6	0.0608 (8)	0.0557 (8)	0.0366 (6)	0.0206 (7)	0.0037 (6)	0.0140 (6)
O11	0.0858 (10)	0.0624 (8)	0.0520 (7)	0.0127 (8)	0.0082 (7)	0.0216 (6)
C33	0.0315 (6)	0.0423 (7)	0.0397 (7)	0.0113 (5)	0.0083 (5)	0.0146 (6)
C29	0.0456 (8)	0.0514 (8)	0.0386 (7)	0.0173 (7)	0.0129 (6)	0.0146 (6)
C32	0.0385 (7)	0.0502 (8)	0.0515 (8)	0.0192 (6)	0.0113 (6)	0.0209 (7)
C40	0.0347 (6)	0.0394 (7)	0.0421 (7)	0.0094 (5)	0.0106 (5)	0.0114 (6)
C39	0.0472 (8)	0.0444 (8)	0.0557 (9)	0.0176 (7)	0.0154 (7)	0.0164 (7)
C31	0.0463 (8)	0.0492 (9)	0.0606 (9)	0.0230 (7)	0.0189 (7)	0.0159 (7)
C30	0.0523 (9)	0.0530 (9)	0.0471 (8)	0.0200 (7)	0.0183 (7)	0.0090 (7)
C34	0.0336 (6)	0.0462 (8)	0.0385 (7)	0.0087 (6)	0.0056 (5)	0.0141 (6)
C37	0.0655 (11)	0.0529 (10)	0.0512 (9)	0.0151 (8)	0.0160 (8)	−0.0014 (7)
C36	0.0596 (10)	0.0558 (10)	0.0413 (8)	0.0155 (8)	0.0088 (7)	0.0060 (7)
C38	0.0573 (10)	0.0433 (8)	0.0699 (11)	0.0185 (7)	0.0216 (8)	0.0089 (8)
C27	0.0317 (6)	0.0490 (8)	0.0420 (7)	0.0092 (6)	0.0112 (5)	0.0194 (6)
N3	0.0416 (6)	0.0543 (7)	0.0332 (5)	0.0112 (6)	0.0111 (5)	0.0134 (5)
N4	0.0589 (8)	0.0430 (6)	0.0347 (6)	0.0248 (6)	0.0132 (5)	0.0101 (5)
C23	0.0426 (7)	0.0415 (7)	0.0522 (8)	0.0150 (6)	0.0138 (6)	0.0139 (6)
C21	0.0344 (6)	0.0363 (6)	0.0360 (6)	0.0099 (5)	0.0119 (5)	0.0099 (5)
C20	0.0358 (6)	0.0381 (7)	0.0371 (6)	0.0102 (5)	0.0138 (5)	0.0094 (5)
C15	0.0380 (7)	0.0449 (8)	0.0374 (7)	0.0086 (6)	0.0152 (5)	0.0099 (6)
C22	0.0331 (6)	0.0389 (7)	0.0406 (7)	0.0100 (5)	0.0118 (5)	0.0136 (5)
C19	0.0494 (8)	0.0428 (8)	0.0456 (7)	0.0176 (6)	0.0179 (6)	0.0143 (6)
C24	0.0473 (8)	0.0433 (8)	0.0768 (11)	0.0182 (7)	0.0197 (8)	0.0222 (8)
C25	0.0417 (8)	0.0595 (10)	0.0783 (12)	0.0185 (7)	0.0175 (8)	0.0418 (9)
C26	0.0386 (7)	0.0645 (10)	0.0565 (9)	0.0142 (7)	0.0137 (7)	0.0343 (8)
C18	0.0624 (10)	0.0467 (9)	0.0655 (10)	0.0259 (8)	0.0292 (8)	0.0177 (8)
C17	0.0756 (12)	0.0459 (9)	0.0629 (10)	0.0222 (8)	0.0386 (9)	0.0069 (7)
C16	0.0615 (10)	0.0520 (9)	0.0418 (7)	0.0120 (8)	0.0241 (7)	0.0056 (7)
O9	0.0747 (9)	0.0853 (10)	0.0369 (6)	−0.0009 (8)	0.0153 (6)	0.0135 (6)
O10	0.0567 (8)	0.0507 (7)	0.0871 (10)	0.0111 (6)	0.0171 (7)	0.0099 (7)

### Geometric parameters (Å, º)

**Table:**

Zn1—N2	2.0146 (12)	C33—C32	1.418 (2)
Zn1—N1	2.0274 (11)	C33—C34	1.430 (2)
Zn1—O5	2.1162 (12)	C29—C30	1.360 (2)
Zn1—O3	2.1793 (12)	C29—H29	0.93
Zn1—O7	2.2151 (11)	C32—C31	1.359 (2)
Zn1—O1	2.2775 (12)	C32—H32	0.93
N1—C5	1.3304 (19)	C40—C39	1.412 (2)
N1—C1	1.3372 (17)	C39—C38	1.368 (3)
N2—C8	1.331 (2)	C39—H39	0.93
N2—C12	1.3321 (19)	C31—C30	1.412 (2)
O1—C6	1.2518 (19)	C31—H31	0.93
O2—C6	1.2508 (18)	C30—H30	0.93
O7—C14	1.2507 (19)	C37—C36	1.364 (3)
O3—C7	1.259 (2)	C37—C38	1.398 (3)
O5—C13	1.269 (2)	C37—H37	0.93
O8—C14	1.2430 (19)	C36—H36	0.93
O6—C13	1.232 (2)	C38—H38	0.93
C6—C1	1.519 (2)	C27—N3	1.357 (2)
O4—C7	1.239 (2)	C27—C26	1.411 (2)
C5—C4	1.384 (2)	C27—C22	1.413 (2)
C5—C7	1.5207 (18)	N3—C15	1.355 (2)
C14—C12	1.520 (2)	N3—H3A	0.86
C1—C2	1.386 (2)	N4—C21	1.3206 (18)
C2—C3	1.386 (2)	N4—H4B	0.86
C2—H2	0.93	N4—H4A	0.86
C12—C11	1.385 (2)	C23—C24	1.370 (2)
C8—C9	1.387 (2)	C23—C22	1.414 (2)
C8—C13	1.526 (2)	C23—H23	0.93
C11—C10	1.382 (3)	C21—C20	1.4361 (19)
C11—H11	0.93	C21—C22	1.4382 (19)
C3—C4	1.387 (2)	C20—C15	1.4100 (19)
C3—H3	0.93	C20—C19	1.411 (2)
C4—H4	0.93	C15—C16	1.412 (2)
C9—C10	1.386 (3)	C19—C18	1.361 (2)
C9—H9	0.93	C19—H19	0.93
C10—H10	0.93	C24—C25	1.402 (3)
N5—C40	1.359 (2)	C24—H24	0.93
N5—C28	1.3588 (19)	C25—C26	1.361 (3)
N5—H5A	0.86	C25—H25	0.93
C28—C29	1.409 (2)	C26—H26	0.93
C28—C33	1.4173 (18)	C18—C17	1.405 (3)
C35—C40	1.413 (2)	C18—H18	0.93
C35—C36	1.418 (2)	C17—C16	1.355 (3)
C35—C34	1.430 (2)	C17—H17	0.93
N6—C34	1.3291 (19)	C16—H16	0.93
N6—H6A	0.86	O9—H9A	0.928 (17)
N6—H6B	0.86	O9—H9B	0.890 (16)
O11—H11A	0.900 (17)	O10—H10B	0.948 (17)
O11—H11B	0.903 (16)	O10—H10A	0.940 (17)
			
N2—Zn1—N1	169.12 (4)	C28—C33—C32	117.75 (14)
N2—Zn1—O5	77.62 (5)	C28—C33—C34	118.85 (13)
N1—Zn1—O5	111.49 (5)	C32—C33—C34	123.40 (13)
N2—Zn1—O3	108.72 (5)	C30—C29—C28	120.01 (14)
N1—Zn1—O3	77.03 (4)	C30—C29—H29	120
O5—Zn1—O3	95.55 (5)	C28—C29—H29	120
N2—Zn1—O7	75.67 (5)	C31—C32—C33	121.01 (14)
N1—Zn1—O7	95.48 (4)	C31—C32—H32	119.5
O5—Zn1—O7	153.00 (4)	C33—C32—H32	119.5
O3—Zn1—O7	89.34 (5)	N5—C40—C39	119.33 (13)
N2—Zn1—O1	99.73 (5)	N5—C40—C35	120.47 (13)
N1—Zn1—O1	74.39 (4)	C39—C40—C35	120.20 (14)
O5—Zn1—O1	93.41 (5)	C38—C39—C40	119.78 (16)
O3—Zn1—O1	151.38 (4)	C38—C39—H39	120.1
O7—Zn1—O1	94.87 (5)	C40—C39—H39	120.1
C5—N1—C1	121.08 (12)	C32—C31—C30	120.49 (15)
C5—N1—Zn1	117.51 (9)	C32—C31—H31	119.8
C1—N1—Zn1	121.10 (10)	C30—C31—H31	119.8
C8—N2—C12	122.19 (13)	C29—C30—C31	120.39 (15)
C8—N2—Zn1	117.72 (10)	C29—C30—H30	119.8
C12—N2—Zn1	120.10 (10)	C31—C30—H30	119.8
C6—O1—Zn1	114.35 (10)	N6—C34—C35	121.08 (15)
C14—O7—Zn1	114.84 (10)	N6—C34—C33	120.02 (15)
C7—O3—Zn1	114.70 (9)	C35—C34—C33	118.90 (13)
C13—O5—Zn1	116.07 (10)	C36—C37—C38	120.31 (17)
O2—C6—O1	126.28 (15)	C36—C37—H37	119.8
O2—C6—C1	117.83 (13)	C38—C37—H37	119.8
O1—C6—C1	115.89 (12)	C37—C36—C35	121.13 (16)
N1—C5—C4	121.23 (12)	C37—C36—H36	119.4
N1—C5—C7	114.56 (13)	C35—C36—H36	119.4
C4—C5—C7	124.19 (13)	C39—C38—C37	120.76 (17)
O8—C14—O7	125.98 (15)	C39—C38—H38	119.6
O8—C14—C12	118.02 (14)	C37—C38—H38	119.6
O7—C14—C12	116.00 (13)	N3—C27—C26	119.00 (14)
N1—C1—C2	120.86 (13)	N3—C27—C22	120.76 (13)
N1—C1—C6	113.66 (12)	C26—C27—C22	120.24 (15)
C2—C1—C6	125.46 (12)	C15—N3—C27	122.52 (12)
C3—C2—C1	118.57 (12)	C15—N3—H3A	118.7
C3—C2—H2	120.7	C27—N3—H3A	118.7
C1—C2—H2	120.7	C21—N4—H4B	120
N2—C12—C11	120.49 (15)	C21—N4—H4A	120
N2—C12—C14	113.39 (12)	H4B—N4—H4A	120
C11—C12—C14	126.11 (14)	C24—C23—C22	120.64 (16)
N2—C8—C9	120.14 (15)	C24—C23—H23	119.7
N2—C8—C13	113.60 (13)	C22—C23—H23	119.7
C9—C8—C13	126.22 (15)	N4—C21—C20	119.76 (13)
O4—C7—O3	127.83 (14)	N4—C21—C22	121.71 (13)
O4—C7—C5	116.49 (15)	C20—C21—C22	118.53 (12)
O3—C7—C5	115.66 (13)	C15—C20—C19	118.30 (13)
O6—C13—O5	126.46 (16)	C15—C20—C21	119.08 (13)
O6—C13—C8	118.82 (15)	C19—C20—C21	122.49 (13)
O5—C13—C8	114.70 (14)	N3—C15—C20	120.40 (14)
C10—C11—C12	118.33 (16)	N3—C15—C16	119.69 (14)
C10—C11—H11	120.8	C20—C15—C16	119.90 (15)
C12—C11—H11	120.8	C27—C22—C23	118.21 (13)
C2—C3—C4	119.85 (14)	C27—C22—C21	118.51 (13)
C2—C3—H3	120.1	C23—C22—C21	123.27 (13)
C4—C3—H3	120.1	C18—C19—C20	120.83 (15)
C5—C4—C3	118.40 (14)	C18—C19—H19	119.6
C5—C4—H4	120.8	C20—C19—H19	119.6
C3—C4—H4	120.8	C23—C24—C25	120.27 (17)
C10—C9—C8	118.51 (16)	C23—C24—H24	119.9
C10—C9—H9	120.7	C25—C24—H24	119.9
C8—C9—H9	120.7	C26—C25—C24	120.93 (15)
C11—C10—C9	120.30 (16)	C26—C25—H25	119.5
C11—C10—H10	119.9	C24—C25—H25	119.5
C9—C10—H10	119.9	C25—C26—C27	119.70 (16)
C40—N5—C28	122.46 (12)	C25—C26—H26	120.1
C40—N5—H5A	118.8	C27—C26—H26	120.1
C28—N5—H5A	118.8	C19—C18—C17	120.14 (17)
N5—C28—C29	119.30 (12)	C19—C18—H18	119.9
N5—C28—C33	120.36 (13)	C17—C18—H18	119.9
C29—C28—C33	120.34 (13)	C16—C17—C18	120.86 (16)
C40—C35—C36	117.82 (15)	C16—C17—H17	119.6
C40—C35—C34	118.93 (14)	C18—C17—H17	119.6
C36—C35—C34	123.22 (14)	C17—C16—C15	119.84 (16)
C34—N6—H6A	120	C17—C16—H16	120.1
C34—N6—H6B	120	C15—C16—H16	120.1
H6A—N6—H6B	120	H9A—O9—H9B	110 (2)
H11A—O11—H11B	105 (2)	H10B—O10—H10A	102 (2)
			
N2—Zn1—N1—C5	116.6 (3)	N2—C8—C13—O5	1.1 (2)
O5—Zn1—N1—C5	−97.47 (10)	C9—C8—C13—O5	−176.85 (16)
O3—Zn1—N1—C5	−6.56 (9)	N2—C12—C11—C10	−1.9 (2)
O7—Zn1—N1—C5	81.49 (10)	C14—C12—C11—C10	179.04 (15)
O1—Zn1—N1—C5	175.03 (10)	C1—C2—C3—C4	0.8 (2)
N2—Zn1—N1—C1	−57.0 (3)	N1—C5—C4—C3	−0.2 (2)
O5—Zn1—N1—C1	88.95 (10)	C7—C5—C4—C3	178.49 (14)
O3—Zn1—N1—C1	179.86 (11)	C2—C3—C4—C5	−0.5 (2)
O7—Zn1—N1—C1	−92.09 (10)	N2—C8—C9—C10	−2.0 (3)
O1—Zn1—N1—C1	1.45 (10)	C13—C8—C9—C10	175.78 (16)
N1—Zn1—N2—C8	143.5 (2)	C12—C11—C10—C9	0.9 (3)
O5—Zn1—N2—C8	−4.29 (11)	C8—C9—C10—C11	1.0 (3)
O3—Zn1—N2—C8	−96.02 (11)	C40—N5—C28—C29	179.43 (13)
O7—Zn1—N2—C8	179.67 (12)	C40—N5—C28—C33	−0.5 (2)
O1—Zn1—N2—C8	87.09 (11)	N5—C28—C33—C32	−179.70 (13)
N1—Zn1—N2—C12	−36.3 (3)	C29—C28—C33—C32	0.3 (2)
O5—Zn1—N2—C12	175.94 (12)	N5—C28—C33—C34	0.0 (2)
O3—Zn1—N2—C12	84.21 (11)	C29—C28—C33—C34	−179.92 (13)
O7—Zn1—N2—C12	−0.10 (11)	N5—C28—C29—C30	179.90 (15)
O1—Zn1—N2—C12	−92.68 (11)	C33—C28—C29—C30	−0.1 (2)
N2—Zn1—O1—C6	174.45 (11)	C28—C33—C32—C31	0.3 (2)
N1—Zn1—O1—C6	3.84 (11)	C34—C33—C32—C31	−179.37 (15)
O5—Zn1—O1—C6	−107.54 (12)	C28—N5—C40—C39	179.49 (14)
O3—Zn1—O1—C6	0.61 (18)	C28—N5—C40—C35	−0.2 (2)
O7—Zn1—O1—C6	98.19 (12)	C36—C35—C40—N5	179.45 (15)
N2—Zn1—O7—C14	0.37 (11)	C34—C35—C40—N5	1.4 (2)
N1—Zn1—O7—C14	173.94 (11)	C36—C35—C40—C39	−0.2 (2)
O5—Zn1—O7—C14	−8.18 (19)	C34—C35—C40—C39	−178.29 (14)
O3—Zn1—O7—C14	−109.16 (12)	N5—C40—C39—C38	−179.20 (15)
O1—Zn1—O7—C14	99.19 (12)	C35—C40—C39—C38	0.5 (2)
N2—Zn1—O3—C7	−166.74 (11)	C33—C32—C31—C30	−1.2 (3)
N1—Zn1—O3—C7	3.66 (11)	C28—C29—C30—C31	−0.7 (3)
O5—Zn1—O3—C7	114.47 (11)	C32—C31—C30—C29	1.4 (3)
O7—Zn1—O3—C7	−92.12 (11)	C40—C35—C34—N6	177.36 (14)
O1—Zn1—O3—C7	6.85 (17)	C36—C35—C34—N6	−0.6 (2)
N2—Zn1—O5—C13	4.95 (12)	C40—C35—C34—C33	−1.8 (2)
N1—Zn1—O5—C13	−168.84 (12)	C36—C35—C34—C33	−179.79 (15)
O3—Zn1—O5—C13	112.94 (13)	C28—C33—C34—N6	−178.06 (14)
O7—Zn1—O5—C13	13.4 (2)	C32—C33—C34—N6	1.7 (2)
O1—Zn1—O5—C13	−94.27 (13)	C28—C33—C34—C35	1.2 (2)
Zn1—O1—C6—O2	172.37 (13)	C32—C33—C34—C35	−179.13 (14)
Zn1—O1—C6—C1	−7.72 (16)	C38—C37—C36—C35	0.7 (3)
C1—N1—C5—C4	0.7 (2)	C40—C35—C36—C37	−0.3 (3)
Zn1—N1—C5—C4	−172.91 (11)	C34—C35—C36—C37	177.63 (17)
C1—N1—C5—C7	−178.13 (12)	C40—C39—C38—C37	−0.2 (3)
Zn1—N1—C5—C7	8.29 (15)	C36—C37—C38—C39	−0.4 (3)
Zn1—O7—C14—O8	178.80 (13)	C26—C27—N3—C15	−176.61 (14)
Zn1—O7—C14—C12	−0.54 (17)	C22—C27—N3—C15	3.1 (2)
C5—N1—C1—C2	−0.41 (19)	N4—C21—C20—C15	−176.62 (14)
Zn1—N1—C1—C2	172.94 (10)	C22—C21—C20—C15	4.3 (2)
C5—N1—C1—C6	−178.99 (11)	N4—C21—C20—C19	7.6 (2)
Zn1—N1—C1—C6	−5.63 (15)	C22—C21—C20—C19	−171.54 (13)
O2—C6—C1—N1	−171.15 (13)	C27—N3—C15—C20	−2.7 (2)
O1—C6—C1—N1	8.94 (18)	C27—N3—C15—C16	176.26 (14)
O2—C6—C1—C2	10.4 (2)	C19—C20—C15—N3	174.83 (14)
O1—C6—C1—C2	−169.56 (14)	C21—C20—C15—N3	−1.1 (2)
N1—C1—C2—C3	−0.3 (2)	C19—C20—C15—C16	−4.1 (2)
C6—C1—C2—C3	178.07 (13)	C21—C20—C15—C16	179.95 (14)
C8—N2—C12—C11	0.9 (2)	N3—C27—C22—C23	179.63 (13)
Zn1—N2—C12—C11	−179.33 (12)	C26—C27—C22—C23	−0.6 (2)
C8—N2—C12—C14	−179.89 (13)	N3—C27—C22—C21	0.2 (2)
Zn1—N2—C12—C14	−0.14 (16)	C26—C27—C22—C21	179.93 (13)
O8—C14—C12—N2	−178.93 (14)	C24—C23—C22—C27	1.3 (2)
O7—C14—C12—N2	0.47 (19)	C24—C23—C22—C21	−179.26 (15)
O8—C14—C12—C11	0.2 (2)	N4—C21—C22—C27	177.11 (13)
O7—C14—C12—C11	179.61 (15)	C20—C21—C22—C27	−3.8 (2)
C12—N2—C8—C9	1.1 (2)	N4—C21—C22—C23	−2.3 (2)
Zn1—N2—C8—C9	−178.67 (12)	C20—C21—C22—C23	176.81 (13)
C12—N2—C8—C13	−176.99 (13)	C15—C20—C19—C18	2.5 (2)
Zn1—N2—C8—C13	3.25 (17)	C21—C20—C19—C18	178.36 (15)
Zn1—O3—C7—O4	−178.96 (15)	C22—C23—C24—C25	−0.9 (3)
Zn1—O3—C7—C5	−0.54 (17)	C23—C24—C25—C26	−0.3 (3)
N1—C5—C7—O4	173.70 (14)	C24—C25—C26—C27	1.0 (2)
C4—C5—C7—O4	−5.1 (2)	N3—C27—C26—C25	179.23 (14)
N1—C5—C7—O3	−4.91 (19)	C22—C27—C26—C25	−0.5 (2)
C4—C5—C7—O3	176.33 (14)	C20—C19—C18—C17	0.6 (3)
Zn1—O5—C13—O6	177.06 (14)	C19—C18—C17—C16	−2.2 (3)
Zn1—O5—C13—C8	−4.65 (18)	C18—C17—C16—C15	0.6 (3)
N2—C8—C13—O6	179.52 (15)	N3—C15—C16—C17	−176.35 (16)
C9—C8—C13—O6	1.6 (3)	C20—C15—C16—C17	2.6 (2)

### Hydrogen-bond geometry (Å, º)

**Table:**

*D*—H···*A*	*D*—H	H···*A*	*D*···*A*	*D*—H···*A*
N3—H3*A*···O9	0.86	1.89	2.7013 (18)	157
N4—H4*A*···O8^i^	0.86	1.98	2.8005 (18)	160
N4—H4*B*···O3^ii^	0.86	2.21	2.9589 (19)	145
N5—H5*A*···O4	0.86	1.88	2.7351 (19)	174
N6—H6*A*···O2^iii^	0.86	2.21	2.9763 (18)	148
N6—H6*B*···O11	0.86	2.10	2.899 (2)	154
O9—H9*A*···O8^iv^	0.93 (3)	1.85 (3)	2.768 (2)	170 (2)
O9—H9*B*···O10	0.89 (2)	1.86 (2)	2.745 (2)	173 (2)
O10—H10*A*···O2^v^	0.94 (2)	1.91 (2)	2.838 (2)	175 (2)
O10—H10*B*···O2	0.95 (3)	1.91 (3)	2.830 (2)	161 (2)
O11—H11*A*···O6^vi^	0.90 (3)	1.93 (3)	2.825 (2)	176 (3)
O11—H11*B*···O1^iii^	0.90 (2)	1.99 (2)	2.8869 (19)	174 (2)

Symmetry codes: (i) *x*, *y*−1, *z*−1; (ii) −*x*+1, −*y*+1, −*z*; (iii) *x*−1, *y*, *z*−1; (iv) −*x*+1, −*y*+1, −*z*+1; (v) −*x*+2, −*y*+1, −*z*+1; (vi) −*x*+1, −*y*+2, −*z*.
